# Distinct Coastal Microbiome Populations Associated With Autochthonous- and Allochthonous-Like Dissolved Organic Matter

**DOI:** 10.3389/fmicb.2019.02579

**Published:** 2019-11-07

**Authors:** Elias Broman, Eero Asmala, Jacob Carstensen, Jarone Pinhassi, Mark Dopson

**Affiliations:** ^1^Centre for Ecology and Evolution in Microbial Model Systems, Linnaeus University, Kalmar, Sweden; ^2^Department of Ecology, Environment and Plant Sciences, Stockholm University, Stockholm, Sweden; ^3^Tvärminne Zoological Station, University of Helsinki, Hanko, Finland; ^4^Department of Bioscience, Aarhus University, Roskilde, Denmark

**Keywords:** 16S rRNA gene, DOM, estuarial and coastal areas, DNA, water

## Abstract

Coastal zones are important transitional areas between the land and sea, where both terrestrial and phytoplankton supplied dissolved organic matter (DOM) are respired or transformed. As climate change is expected to increase river discharge and water temperatures, DOM from both allochthonous and autochthonous sources is projected to increase. As these transformations are largely regulated by bacteria, we analyzed microbial community structure data in relation to a 6-month long time-series dataset of DOM characteristics from Roskilde Fjord and adjacent streams, Denmark. The results showed that the microbial community composition in the outer estuary (closer to the sea) was largely associated with salinity and nutrients, while the inner estuary formed two clusters linked to either nutrients plus allochthonous DOM or autochthonous DOM characteristics. In contrast, the microbial community composition in the streams was found to be mainly associated with allochthonous DOM characteristics. A general pattern across the land-to-sea interface was that Betaproteobacteria were strongly associated with humic-like DOM [operational taxonomic units (OTUs) belonging to family Comamonadaceae], while distinct populations were instead associated with nutrients or abiotic variables such as temperature (Cyanobacteria genus *Synechococcus*) and salinity (Actinobacteria family Microbacteriaceae). Furthermore, there was a stark shift in the relative abundance of OTUs between stream and marine stations. This indicates that as DOM travels through the land-to-sea interface, different bacterial guilds continuously degrade it.

## Introduction

Dissolved organic matter (DOM) is the largest pool of organic carbon in the global oceans (∼660 pg C) and it is up to 200-fold greater than that in organic particles or marine life (Hansell et al., 2009; Jiao et al., 2010). Rivers and streams discharge large quantities of terrestrial organic matter (denoted allochthonous organic matter; 0.9 pg C per year) that directly influences coastal ecosystems (Cole et al., 2007). A fraction of DOM absorbs light, and the optical characteristics of this colored DOM (CDOM) are widely used in studying the origin and fate of the DOM pool in aquatic systems (Massicotte et al., 2017). In addition to organic matter, rivers also deliver nutrients including nitrogen and phosphorus compounds from the catchment to the coastal environment promoting phytoplankton growth (Conley et al., 2009). In combination with the ongoing increase in global ocean surface temperature (Rhein et al., 2013) and estimated increase in riverine discharge for one-third of the land surface (van Vliet et al., 2013), climate change is likely to cause increased inputs of terrestrial organic matter (Kritzberg and Ekström, 2012) and nutrients into estuaries and other coastal waters.

*In situ* biological production of DOM (i.e., autochthonous production) is derived from phytoplankton photosynthesis, incomplete grazing of phytoplankton, and viral lysis or death of bacterial cells (Thornton, 2014). The amount of autochthonous DOM is therefore especially high in shallow coastal systems rich in both pelagic and benthic primary producers, such as eutrophicated estuaries (Markager et al., 2011; Asmala et al., 2018b). Compared to allochthonous DOM, the biological DOM produced *in situ* is typically considered to have a lower molecular weight and constitutes a labile carbon source for heterotrophic microbes (Jiao et al., 2010). This labile autochthonous organic matter is rapidly metabolized by heterotrophic bacteria (Hansell, 2013; Asmala et al., 2018a), and the organic matter pool in the marine environment is eventually turned into refractory DOM that can last for millennia (Jiao et al., 2010). Heterotrophic bacteria are important degraders of DOM (Tranvik, 1992, 1998), and a large portion of this degradation results in the release of CO_2_ (Del Giorgio and Cole, 1998; Fasching et al., 2014). Considering that the surface temperature in the global oceans is increasing (Rhein et al., 2013) and this will enhance algal blooms (Beaulieu et al., 2013), climate change is likely to increase the production of autochthonous DOM in coastal systems and microbial populations associated with degradation of this carbon source.

Microbial degradation of DOM is an essential process in carbon cycling and the use of modern molecular tools has helped to elucidate the link between microbial communities and autochthonous/allochthonous DOM. Many worldwide studies have been conducted in the laboratory using amendment of organic matter. For example, Rocker et al. (2012) found that Alpha- and Gammaproteobacteria in marine and estuarine water became dominant when supplied with humic acids. Mesocosm experiments containing Baltic Sea coastal water supplied with soil extracted DOM stimulated growth of, e.g., Bacteroidetes, Alpha-, and Betaproteobacteria (Traving et al., 2017). A similar controlled experiment of riverine freshwater showed that only a subset of the microbial populations were able to degrade terrestrial derived DOM that had a high molecular weight (Logue et al., 2015). In addition, a time-series study conducted on the eastern coast of Uruguay showed that Alphaproteobacteria were associated with low molecular weight humic-like DOM; Bacteroidetes and Gammaproteobacteria were associated with high molecular weight humic-like DOM; and Betaproteobacteria were linked to both autochthonous and allochthonous DOM (Amaral et al., 2016). This change in the microbial community composition upon degradation of different DOM has been confirmed in other laboratory experiments (e.g., Cottrell and Kirchman, 2000; Judd et al., 2006). Studies based on *in situ* field microbial communities are scarcer but have shown an indication of congruent results with laboratory studies. For example, the diversity of the 16S rRNA gene-based microbial community in freshwater from Canada was found to be associated with the quantity and optical characteristics of the DOM pool (Ruiz-González et al., 2015), even though specific populations associated with the different DOM pools were not reported. Roiha et al. (2016) used CDOM and fluorescent dissolved organic matter (FDOM) data to determine the origin of DOM in a freshwater alpine region. The results showed that different subarctic bacterial guilds were associated with either terrestrial (allochthonous) or algal (autochthonous) carbon compounds (Roiha et al., 2016). Microbial community structures and diversity have been studied in estuaries (e.g., Bernhard et al., 2005; Campbell and Kirchman, 2013) and recently with the use of DOM molecular composition using mass spectrometry (Osterholz et al., 2018). However, the specific microbial populations in estuaries and adjacent streams associated with allochthonous- or autochthonous-like DOM have not been investigated using modern sequencing tools and a comprehensive dataset of CDOM and FDOM variables.

The aim of this study was to investigate changes in microbial community composition in the land-to-sea interface and its association with allochthonous or autochthonous DOM. We hypothesized that (1) microbial populations associated with humic DOM would decrease along the land-to-sea interface and (2) the microbial community structure in coastal water closer to land, rather than sea, would be influenced by both allochthonous and autochthonous DOM. To answer these questions we analyzed a large dataset of optical DOM variables (Asmala et al., 2018b) with high-throughput 16S rRNA gene sequencing data collected over 6 months across the freshwater–estuarine salinity gradient in Roskilde Fjord (Denmark), as well as five adjacent streams.

## Materials and Methods

### Field Sampling

**FIGURE 1:**
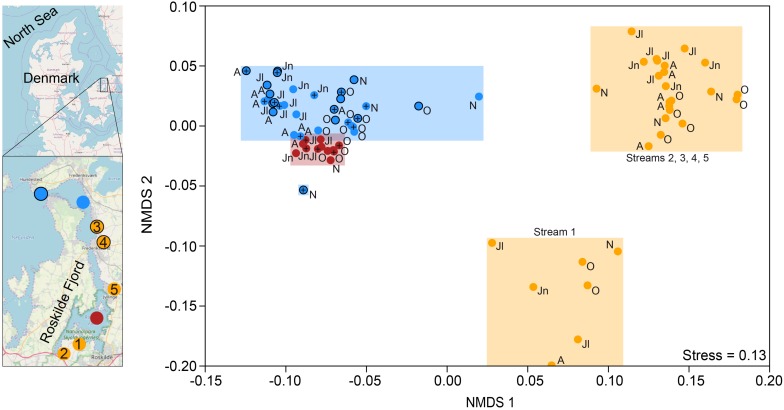
Map of Roskilde Fjord, the sampling stations, and a NMDS based on Bray–Curtis dissimilarity of all OTUs. The sites were grouped as “inner estuary” (marine station 1; red dots), “outer estuary” (marine stations 2 and 3; blue dots and blue dots with a black border, respectively), and streams (all five stream sites; yellow dots). Each dot in the NMDS denotes a sampling occasion. For the marine stations, open symbols denote samples taken at 1 m depth while the cross symbols denote samples taken at 4 m depth. Observations are labeled with their month of sampling: August (A), June (Jn), July (Jl), October (O), and November (N). Three streams discharge to the inner estuary (1-Boserup, 2-Lejre, and 5-Vaerebro) and two streams discharge to the outer estuary (3-Havelse and 4-Langsø). The map layer is OpenStreetMap©.

Roskilde Fjord is a shallow estuary with a mean depth of 3 m and an average freshwater residence time of 8 months in the inner broad of the estuary (Kamp-Nielsen, 1992). Water from Roskilde Fjord was sampled once or twice per month between 10 June and 22 November 2014 (full details in Asmala et al., 2018b). In brief, 5 L water was collected from three marine stations at depths of 1 and 4 m. Station 1 was located in the inner part of the estuary, close to the town of Roskilde (i.e., inner estuary; *n* = 12), station 2 was located in the outer estuary, and station 3 was located at the mouth to the larger Isefjord (Figure 1; *n* = 16 for each station). In addition to the three sampling sites in the estuary, 5 L of the surface water from five streams was also sampled during the same period. Measurements of pH, temperature, salinity, and conductivity were conducted *in situ* during the sampling campaign (results and methodology presented in Asmala et al., 2018b). Water from each collected sample was divided for DNA extraction and chemistry/optical measurements.

A detailed description of sample preparation, methodology, instruments, chemistry, and optical variables is found in Asmala et al. (2018b). Abiotic variables were analyzed from the same water samples used for DNA extraction. In brief, nutrients [total nitrogen (TN) and phosphorus, total dissolved nitrogen and phosphorus, and dissolved inorganic nitrogen (DIN) and phosphorus] were measured spectrophotometrically according to Hansen and Koroleff (1999), DOC concentrations were measured on a Shimadzu TOC-V_CPH_ analyzer, CDOM absorption on a Shimadzu 2401PC spectrophotometer, and FDOM excitation–emission matrices on a Varian Cary Eclipse fluorometer (Agilent). CDOM variables used in this study included absorption coefficients at 254 and 400 nm (i.e., optical DOM density: *a*_(CDOM__254__)_ and *a*_(CDOM__400__)_, respectively), specific ultraviolet absorbance of DOM (i.e., color intensity, SUVA_254_), and absorption spectral slopes between 275 and 295 plus 350 and 400 nm (“low molecular weight/decreasing aromaticity” indicators: S_275__–__295_ and S_350__–__400_, respectively). SUVA_254_ can be used as a proxy for aromaticity (Weishaar et al., 2003) and the extent of biogeochemical processing of the DOM pool (Massicotte et al., 2017). FDOM variables included protein-like labile indicator “peak T,” the biological index BIX (i.e., autochthonous origin), humic-like indicator “peak C,” and the humification index HIX (i.e., allochthonous origin) (Coble, 1996; Zsolnay et al., 1999). Asmala et al. (2018b) conducted parallel factor analysis (PARAFAC; Stedmon et al., 2003) of the FDOM data and found that DOM characteristics (i.e., protein-like or humic-like) clustered the samples into three distinct groups: “inner estuary” (marine station 1), “outer estuary” (marine stations 2 and 3), and “streams” (the five stream stations). See Supplementary Table S1 for a summary of the chemical and optical data, and Figure 1 for an overview of the stations and streams. In this study, this grouping was retained for further analyses with regard to microbial community composition and its correlation to the abiotic data.

### DNA Extraction, Sequencing, and Bioinformatics

Each sample was filtered through one 0.22 μm Supor-200 25 mm filter (PALL Corporation; 500 mL for each marine sample and 100 mL for each stream) and placed in sterile 2 mL cryogenic tubes (Nalgene, ThermoFisher Scientific) containing 1× TE buffer (Tris and EDTA, pH 8.0). The frozen filters were then stored at −80°C. DNA was extracted from the frozen water filters with the PowerWater DNA kit (MO BIO Laboratories). Extracted DNA was stored at −20°C until 16S rRNA gene amplification and Illumina library preparation according to Lindh et al. (2015) using PCR primers 341f and 805r (spanning regions V3–V4) (Herlemann et al., 2011) with no additional modification for SAR11 (Apprill et al., 2015), and Nextera indexes for multiplexing with a modified PCR program as described by Hugerth et al. (2014). The library was sequenced on the Illumina MiSeq platform with a 2 × 300 bp pair-end setup at Science for Life Laboratory (SciLifeLab), Stockholm. Illumina sequencing yielded 5999–175,788 sequences from the stream samples and 11,692–302,691 sequences from the marine water. The number of reads obtained after sequencing, merging of pair-ends, and quality trimming as well as the amount of clustered operational taxonomic units (OTUs) are available in Supplementary Table S2.

The 16S rRNA gene sequences were analyzed according to the UPARSE pipeline (Edgar, 2013) with a 97% OTU clustering sequence similarity and 95% identity threshold against the small-subunit Ref NR 99 SILVA version 123 database (Quast et al., 2013). The final OTU tables were analyzed using the software Explicet (Robertson et al., 2013). On average, 1779 OTUs could be clustered for the marine water samples and 2591 for the stream samples (Supplementary Table S2). A full list of OTUs and relative abundance (*x*/sum × 100) is available in Supplementary Table S3. OTUs classified as chloroplasts were excluded from the final dataset. Rarefaction analysis of OTUs versus read counts showed that a large percentage of the microbial communities had been sequenced, but a portion remain to be discovered (Supplementary Figure S1). As unclassified OTUs belonging to the Betaproteobacteria family Comamonadaceae was the major bacterial group associated with humic-like DOM (especially top abundant OTUs with numbers 66 and 103), these sequences were annotated with BLAST against the NT database with default settings at NCBI’s website (date accessed 2019-09-21).

### Statistics and Correlations Between Microbial Taxa and Abiotic Factors

SPSS was used to construct Spearman’s rank correlation matrixes to find associations with DOM characteristics and microbial populations. Shannon *H*’s alpha diversity index was calculated based on OTU level after sub-sampling to the lowest sample size (2821 annotated reads) and bootstrapping 100 times. The software Past 3.25 (Hammer et al., 2001) was used to conduct non-metric multidimensional scaling (NMDS) of Bray–Curtis dissimilarities, CCAs based on the relative abundance 16S rRNA gene and/or chemistry, and PERMANOVA tests (9999 permutations) of NMDS groups (i.e., stations) and CCA axes (significance of constraints test). The R package vegan (Oksanen et al., 2018) was used with default settings to construct CCAs based on all OTUs and samples with missing abiotic data were removed from the analyses (e.g., when combining data from all three systems). Shapiro–Wilk tests were used to test for assumption of normally distrusted data. As the data were not normally distributed, statistical tests for alpha diversity were conducted with analysis of covariance (ANCOVA) with a bootstrap × 1000, using Shannon’s *H* index as a dependent variable, the stations as an independent factor and the time of sampling (month) as a covariate. Tests between stations for alpha diversity and community composition were conducted with non-parametric Kruskal-Wallis tests and Spearman correlations as assumptions for normally distributed data could not be met (Shapiro–Wilk test). Spearman correlations were visualized as networks with a *p* < 0.05 and rho (*r*_s_) >0.7 or <−0.7 in Cytoscape 3.5.1 (Shannon et al., 2003). To try to decouple the influence of the abiotic variables from each other, multiple linear regression (with bootstrap × 1000) was conducted in SPSS with taxonomic data as dependent variables (abundant phyla or Proteobacteria class) and the abiotic data as independent variables.

### Data Availability

The raw sequence data have been uploaded to the NCBI database with the BioProject id: PRJNA396662.

## Results

### Results From High-Throughput 16S rRNA Gene Amplicon Sequencing

Non-metric multidimensional scaling ordination based on Bray–Curtis dissimilarity showed that the inner estuary had a microbial community composition that was significantly different from the outer estuary (PERMANOVA, *p* < 0.01; Figure 1). The microbial community composition in the five studied streams was significantly different when compared to the estuarine community (PERMANOVA, *p* < 0.01; Figure 1). Furthermore, the community composition in one stream (1-Boserup) was markedly different from the others (Figure 1).

Analysis of covariance with bootstrap × 1000 (with month as a covariate) of Shannon’s *H* alpha diversity in the marine stations showed that there was difference between stations (*F* = 4.8, *p* < 0.05) and the time of sampling (*F* = 22.5, *p* < 0.01) were statistically significant. In more detail, alpha diversity in the estuary was significantly lower between marine stations 1 and 2 compared to the boundary station 3 (Shannon’s *H* index 5.4 ± 0.8 and 5.5 ± 1.0 compared to 6.0 ± 0.8, *p* < 0.05; Kruskal–Wallis test; mean ± one standard deviation; Supplementary Table S4). The alpha diversity was also significantly lower during summer (5.2 ± 0.5, *n* = 21; June–August) compared to autumn (6.2 ± 0.8, *n* = 20; October and November; *p* < 0.01; Supplementary Table S4). ANCOVA analysis of the streams showed that there was no difference between the stations but the time of sampling (month) was statistically significant (6.3 ± 1.1, *n* = 29; *F* = 8.5, *p* < 0.01; Supplementary Table S4).

### Microbial Community Structure in Roskilde Fjord

**FIGURE 2:**
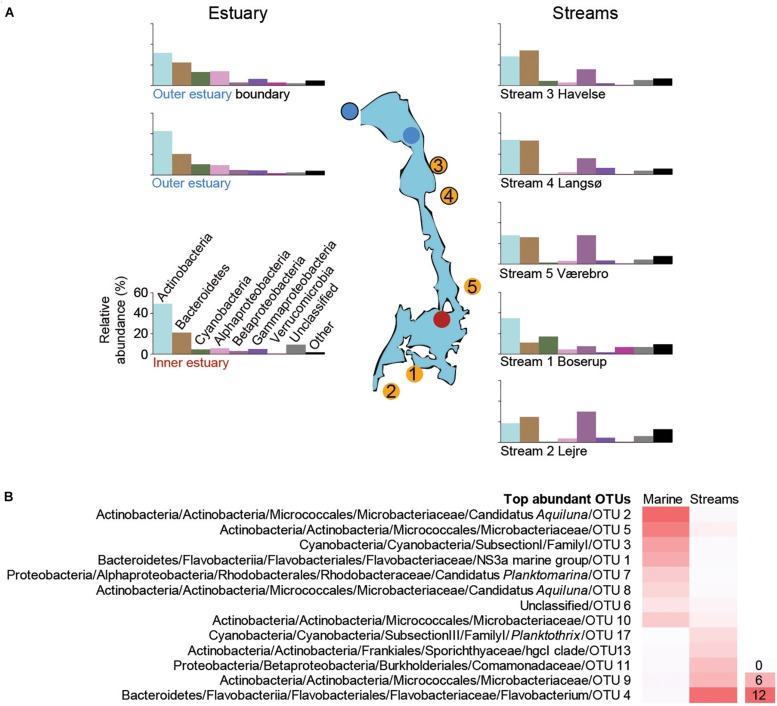
**(A)** The most abundant taxonomic groups (phyla and Proteobacteria classes) based on the 16S rRNA gene data. Bars are averages from each sampling site over the time series (1 m depth data were used for the marine stations). Data from individual samples from all time points are shown in Supplementary Figures S2 (marine), S4 (streams). The *y*-axis scale is the same for all bar charts and is shown next to the lower left bar chart. **(B)** Heatmap of the most abundant OTUs (>0.2% average between aquatic systems) that were present in both marine and stream stations. For each OTU the lowest taxonomic classification is shown followed by the unique OTU number, while the color gradient shows the average relative abundance (%) for all marine or stream stations.

The marine sites (1 m depth data) were dominated by the phyla Actinobacteria, Bacteroidetes, Cyanobacteria, Alpha-, Beta-, and Gammaproteobacteria across all sampling occasions (*n* = 44; Figure 2A and Supplementary Figure S2). Verrucomicrobia were more common at the mouth of Roskilde Fjord compared to stations 1 and 2 (Figure 2A; Kruskal–Wallis test, *p* < 0.05), whereas the phylum Actinobacteria had significantly lower relative abundance at marine station 3 compared to stations 1 and 2 (Kruskal–Wallis test; *p* < 0.01; Figure 2A). Alphaproteobacteria had a significantly lower relative abundance at station 1 compared to stations 2 and 3 (Kruskal–Wallis test; *p* < 0.01; Figure 2A). The relative abundance of Cyanobacteria was high during summer and declined in autumn at all three stations (Supplementary Figure S2) with a significant difference between summer and autumn months (*p* < 0.05 for all three stations).

The most abundant OTUs in the marine water were similar at 1 and 4 m depths, indicating that the water column was mixed (Supplementary Figure S3). A large part of the relative abundance was shared among four OTUs belonging to the Actinobacteria family Microbacteriaceae (together reaching 65.6% during June) with the most abundant OTU aligning to the candidate genus *Aquiluna* that reached 21.8% in August (Supplementary Figure S3). The increase in relative abundance of Cyanobacteria during summer was mainly associated with a single unclassified OTU belonging to the Subsection I family I that had a relative abundance up to 48.3% in station 3 (Supplementary Figure S3). The relative abundance of Bacteroidetes was mainly represented by one OTU belonging to the family Flavobacteriaceae that reached up to 23.1% during August. In contrast to the most abundant Cyanobacteria OTUs, the Bacteroidetes decreased in summer while increasing during autumn (Supplementary Figure S3). The most abundant OTU in the Alphaproteobacteria class belonged to the candidate genus *Planktomarina* within the Rhodobacteraceae that increased from <5% to >10% during August and October before declining again in November (Supplementary Figure S3).

### Microbial Community Structure in the Streams

All five streams were dominated by Actinobacteria, Bacteroidetes, Beta-, and Gammaproteobacteria (1-Boserup, *n* = 7; 2-Lejre, *n* = 7; 3-Havelse, *n* = 7; 4-Langsø, *n* = 4; and 5-Vaerebro, *n* = 4; Figure 2A and Supplementary Figure S4). Stream 1-Boserup (closest to the town of Roskilde) had a significantly higher relative abundance of Cyanobacteria and Verrucomicrobia compared to the other four streams (Kruskal–Wallis test; *p* < 0.01; Figure 2A). In addition, stream 2-Lejre situated further inland had a significantly higher relative abundance of Epsilonproteobacteria compared to the other four streams (Kruskal–Wallis test; *p* < 0.05; Figure 2A). Moreover, a significantly lower relative abundance of Betaproteobacteria was found during summer (13.9 ± 7.2%, *n* = 17; June–August) compared to autumn (25.3 ± 14.8%, *n* = 12; October and November; *p* < 0.01; Supplementary Figure S4). The opposite trend was observed for Bacteroidetes that had a significantly (*p* < 0.01; Supplementary Figure S4) higher relative abundance during summer (35.9 ± 10.3%; *n* = 13) in streams 2–5 compared to autumn (18.7 ± 7.8%; *n* = 9).

Focusing on the most abundant OTUs in the streams, one Flavobacterium OTU was responsible for the increase in Bacteroidetes abundance during summer in streams 2–5 (Supplementary Figure S5). Two different genera of Actinobacteria were abundant. These included three OTUs belonging to the family Sporichthyaceae mainly present in stream 1-Boserup (up to 18.9% in August) and OTUs affiliated with the Microbacteriaceae family mainly present in streams 2–5 (up to 24.6% in July; Supplementary Figure S5). The high abundance of Cyanobacteria during summer in stream 1-Boserup was mainly due to a single OTU belonging to the Subsection III family I genus *Planktothrix* (up to 56.6% in August). Abundant Betaproteobacteria OTUs mainly belonged to the family Comamonadaceae in streams 2–5 (e.g., 29.2% for one OTU in October) and one OTU belonging to the genus *Polynucleobacter* in stream 1-Boserup (0.6–2.3%; Supplementary Figure S5). Finally, OTUs present in both the streams and marine stations showed a stark contrast in relative abundance, even though they belonged to similar taxonomic groups, between the two aquatic systems (Figure 2B and Supplementary Table S3).

### Associations Between Microbial Groups and Abiotic Variables

**FIGURE 3:**
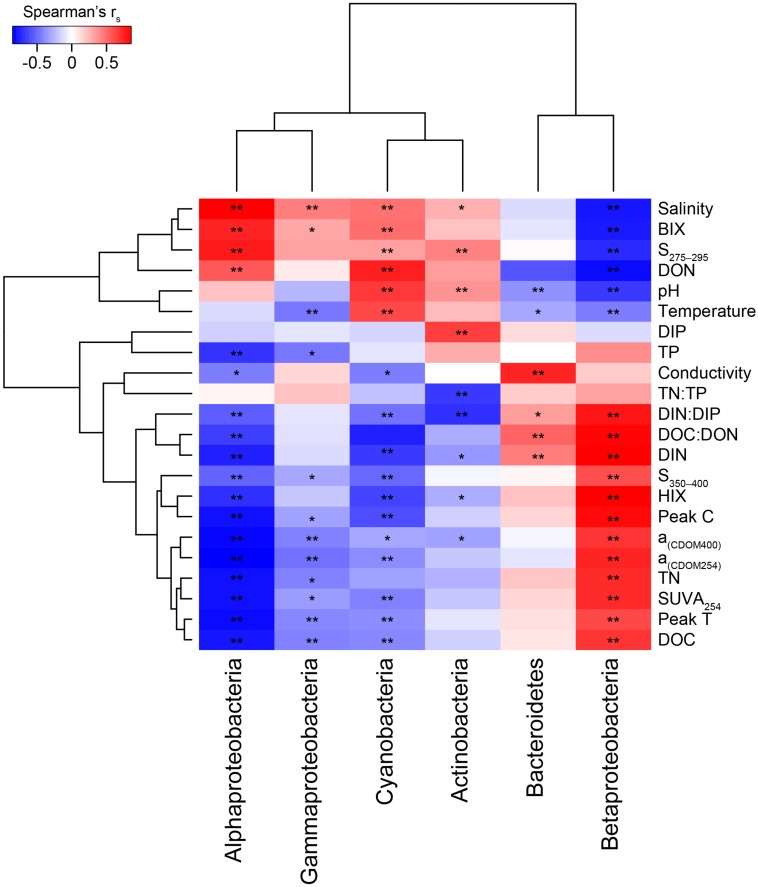
Two clustered heatmap (taxa and abiotic variables) showing Spearman’s correlation *r*_s_ values between the most abundant phyla plus Proteobacteria classes and the abiotic factors. The stars denote ^∗^*p* < 0.05 and ^∗∗^*p* < 0.01. Chemistry abbreviations: DIN and DIP, dissolved inorganic nitrogen and phosphorus; DON and DOC, dissolved organic nitrogen and carbon; and TN and TP, total nitrogen and phosphorus. Optical parameters were CDOM aromaticity indicators *a*_(CDOM__254__)_, *a*_(CDOM__400__)_, and SUVA_254_; spectral slopes for “low molecular weight/decreasing aromaticity” indicators S_275–295_ and S_350–400_; humic-like FDOM indicators peak C and the humification index HIX; and the autochthonous-like FDOM indicators peak T and the biological index BIX.

To identify possible associations between microbial community composition and abiotic factors, we investigated the distribution of microbes at different levels of taxonomic resolution to chemical and optical characteristics previously reported from the same samples (Asmala et al., 2018b). Briefly, Asmala et al. (2018b) showed that the streams were richer in TN, DIN, and DOC when compared to the estuary (Supplementary Table S1A). In addition, the DOM in the streams exhibited pronounced terrestrial-like features such as high SUVA_254_, peak C, and low S_275__–__295_ values that indicated high aromaticity and large molecular size of the DOM molecules (Supplementary Table S1B; Asmala et al., 2018b). The DOC concentration, *a*_(CDOM__254__)_, FDOM peak C, and HIX were higher in the inner versus outer estuary indicating increased humic-like DOM at stations more influenced by inputs from land (Supplementary Table S1B; see Figure 3 and methods for explanation of optical variables) (Asmala et al., 2018b).

When correlating abundant phyla from the whole dataset (i.e., streams and marine stations), Actinobacteria had a weak positive correlation with salinity and S_275__–__295_ (*p* < 0.05; *r*_s_ = 0.41 and 0.26, respectively) and negative correlations with CDOM characteristics and HIX (*p* < 0.05; *r*_s_ = −0.31 and −0.28, respectively; Figure 3). This indicated an association with the salinity gradient in the estuary and a negative relationship with humic-like DOM. Furthermore, Bacteroidetes correlated with pH (*p* < 0.05; *r*_s_ = −0.3), while Cyanobacteria correlated positively with temperature and BIX (*p* < 0.01; *r*_s_ = 0.61 and 0.49, respectively) and negatively with DOC and many of the CDOM and FDOM variables, e.g., *a*_(CDOM__254__)_, *a*_(CDOM__400__)_, peak C, and HIX (*p* < 0.05; *r*_s_ < −0.3; Figure 3). This indicated an association between Cyanobacteria and warmer surface water plus primary production of autochthonous DOM. Similar results, i.e., negative correlations for DOC and many of the CDOM and FDOM variables, were also found for Alpha- and Gammaproteobacteria (*p* < 0.05; *r*_s_ < −0.3; Figure 3). In contrast, Betaproteobacteria correlated positively with the CDOM and FDOM variables for humic-like DOM (*p* < 0.01; *r*_s_ > 0.7; Figure 3) and negatively with S_275__–__295_ (*p* < 0.01; *r*_s_ = −0.71; Supplementary Table S5). These results indicated an association between Betaproteobacteria and humic-like DOM. Multiple regression analyses between abundant phyla and Proteobacteria classes showed that Betaproteobacteria was significantly associated with the humic indicator HIX index (*p* = 0.019; Table 1), while Actinobacteria was associated with, e.g., salinity (*p* = 0.015) and Cyanobacteria with temperature (*p* = 0.021; Table 1).

**TABLE 1:** Multiple linear regression (bootstrap × 1000) with the most abundant phyla plus Proteobacteria classes and selected abiotic factors that change throughout the estuary system (salinity, pH, temperature, DOC) plus the optical FDOM indexes HIX and BIX.

	**Actinobacteria**	**Bacteroidetes**	**Cyanobacteria**	**Alpha**	**Beta**	**Gamma**
Temperature	NS	NS	0.021^∗^	0.001^∗∗^	NS	NS
pH	NS	NS	0.001^∗∗^	NS	0.004^∗∗^	NS
Salinity	0.015^∗^	NS	NS	NS	NS	NS
DIN	NS	NS	NS	NS	NS	NS
DIP	0.008^∗∗^	NS	NS	0.040^∗^	NS	NS
DOC	0.038^∗^	NS	0.002^∗∗^	NS	NS	NS
HIX	NS	NS	NS	NS	0.019^∗^	NS
BIX	NS	NS	NS	NS	NS	NS
*F*_(8,27)_	6.766	3.089	7.838	14.939	25.078	1.715
*R*^2^	0.667	0.478	0.669	0.816	0.881	0.337

*The table shows the model’s *p*-values while the stars denote ^∗^*p* < 0.05 and ^∗∗^*p* < 0.01. NS, non-significant variable.*

**FIGURE 4:**
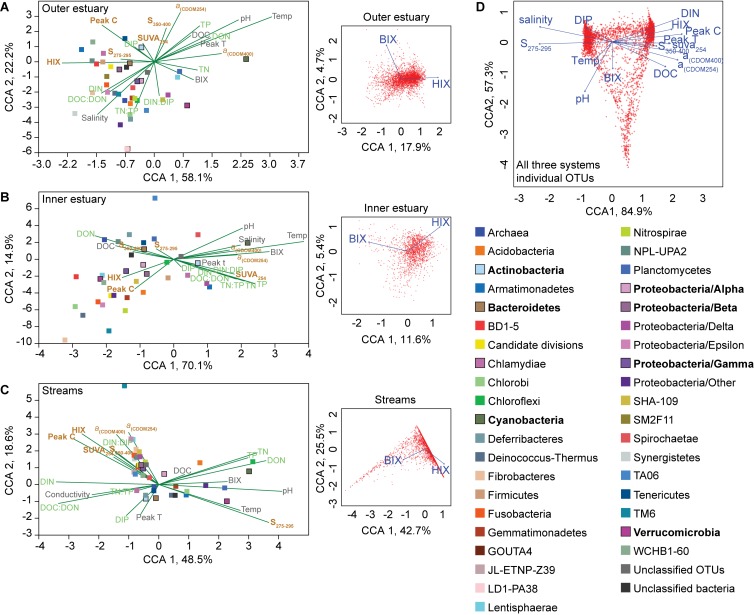
Canonical correspondence analyses (CCAs) for the “outer estuary **(A)**, “inner estuary” **(B)**, and the five streams **(C)**. The CCAs were based on the chemistry/optical data and the relative abundance of phyla (Proteobacteria were divided into classes). The text colors in the CCAs denote abiotic factors (gray) and further division into nutrient-related (green) or DOM characteristics (brown). The insert subgraphs to the right of each main graphs show CCAs based on all OTUs and the BIX and HIX variables as environmental parameters. The color legend on the right side correspond to the taxonomic groups in the main graph CCAs showing different phyla and proteobacteria classes. Taxa with high relative abundance (as shown in Figure 2) are denoted by bold text and bold outlines on the respective square symbols. **(D)** CCA based on all OTUs from all three study systems (streams, inner, and outer estuary). See Figure 3 for abbreviations and explanations of the chemical and optical variables.

Canonical correspondence analyses (CCAs) of phyla and abiotic factors showed that the microbial community composition in the outer estuary was grouped with salinity and nutrients [CCA axis 1, *p* < 0.001 and CCA axis 2, *p* < 0.001; PERMANOVA (9999 permutations); Figure 4A]. While in the inner estuary the phyla were linked to either nutrients or DOM characteristics (no statistical significance for CCA axes; Figure 4B). In contrast, the microbial community composition in the streams was found to be mainly grouped with allochthonous DOM characteristics [CCA axis 1, *P* < 0.001; and CCA axis 2, *p* < 0.05; PERMANOVA (9999 permutations); Figure 4C]. CCAs testing the effect of BIX and HIX on the community composition showed that OTUs in the streams clustered closely with HIX (with CCA axis 1 explaining up to 42.7% of the OTU distribution), while the inner and outer estuary showed no clear clustering with either BIX or HIX (Figure 4). The difference in microbial communities and abiotic variables between the streams and estuary was also indicated with CCA of all OTUs from all three study systems (i.e., streams, inner, and outer estuary; Figure 4D). The CCA formed two clusters of OTUs related to: (1) marine/autochthonous with salinity, temperature, DIP, and “low molecular weight/decreasing aromaticity” indicator S_275__–__295_ and (2) freshwater/allochthonous with DIN, and humic-like DOM characteristics such as HIX, DOC, Peak C, and CDOM (Figure 4D).

**FIGURE 5:**
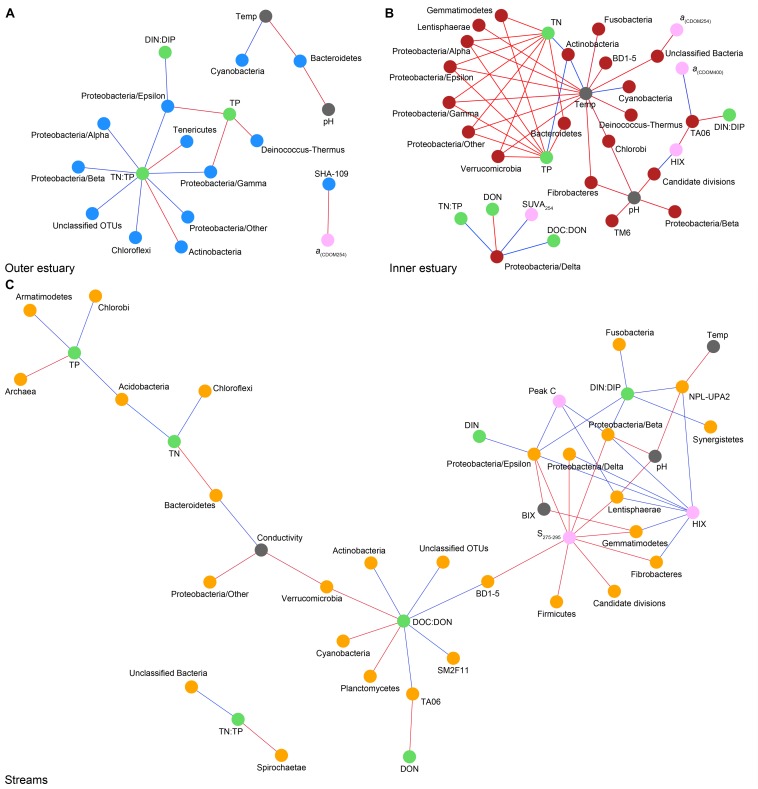
Spearman correlations (visualized as networks) between abiotic variables and phyla with statistically significant correlations (*p* < 0.05) and *r*_s_ > 0.7 (blue lines) or < –0.7 (red lines). For the microbial phyla, blue dots show “outer estuary” **(A)**, red dots denote the “inner estuary” **(B)**, and yellow dots show data from the five stream sites **(C)**. Abiotic variables are marked by gray dots, with green dots for nutrient-related variables and pink dots for CDOM and humic-like-related variables.

**FIGURE 6:**
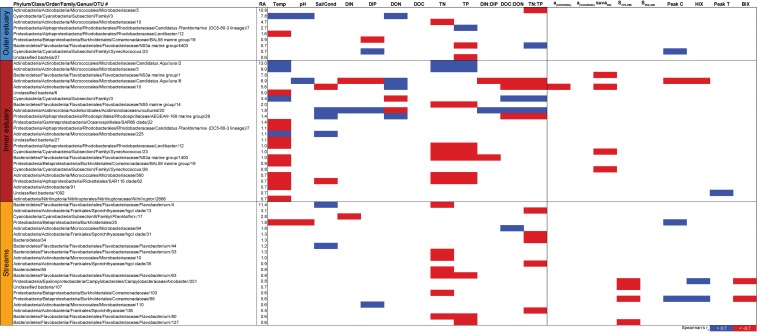
Spearman correlations between abiotic variables, optical variables, and the 30 most abundant OTUs from the outer estuary (marine stations 2 and 3), inner estuary (marine station 1), and all the streams. The colored cells denote statistically significant correlations (*p* < 0.05) with red color denoting Spearman’s *r*_s_ ≤ –0.7, and blue ≥0.7. RA denotes relative abundance (%) of the OTU compared to the whole community.

Spearman’s rank correlations of statistically significant abiotic variables and microbial phyla plus Proteobacteria classes [two-tailed, *p* < 0.05; restricted to rho (*r*_s_) > 0.7 or <−0.7] showed that the “outer estuary,” “inner estuary,” and the “streams” had different characteristics (Figure 5; see Supplementary Table S5 for all correlations). The microbial community composition in the outer estuary was mainly associated with nutrient concentrations and ratios between nutrients. For example, the TN:TP ratio correlated positively with the abundant Alpha-, Beta-, Epsilon-, and Gammaproteobacteria (Figure 5A). Cyanobacteria correlated positively with temperature, while the Actinobacteria correlated negatively with the TN:TP ratio. Interestingly, the abundant phylum Bacteroidetes correlated negatively with both temperature and pH (Figure 5A). In the inner estuary, several phyla and Proteobacteria classes correlated negatively with temperature, TN, and TP including Verrucomicrobia, Alpha-, Epsilon-, and Gammaproteobacteria (Figure 5B). However, Cyanobacteria correlated positively with temperature, as well as the abundant phylum Actinobacteria that also correlated positively with TN and TP. Deltaproteobacteria correlated positively to TN:TP, DOC:DON (dissolved organic carbon/nitrogen, respectively), and SUVA_254_ while negatively to DON (Figure 5B). Looking closer at the top 30 most abundant OTUs with statistically significant correlations, a large proportion of the OTUs correlated with nutrients with only a few associations with optical DOM variables (Figure 6). In more detail, OTU 23 in the outer estuary aligning to the genus *Synechococcus* positively correlated with peak C (humic-like DOM). In the inner estuary, OTUs aligning within Bacteroidetes (family Flavobacteriaceae), Actinobacteria (family Microbacteriaceae), and Cyanobacteria (genus *Synechococcus*) totaled 22.1% of the relative abundance and correlated negatively with CDOM variables (Figure 6). Taken together, these results indicated that microbial populations in the outer estuary had more associations with nutrients (or the ratio of nutrient concentrations), while populations in the inner estuary correlated both with nutrients and optical characteristics of DOM.

**FIGURE 7:**
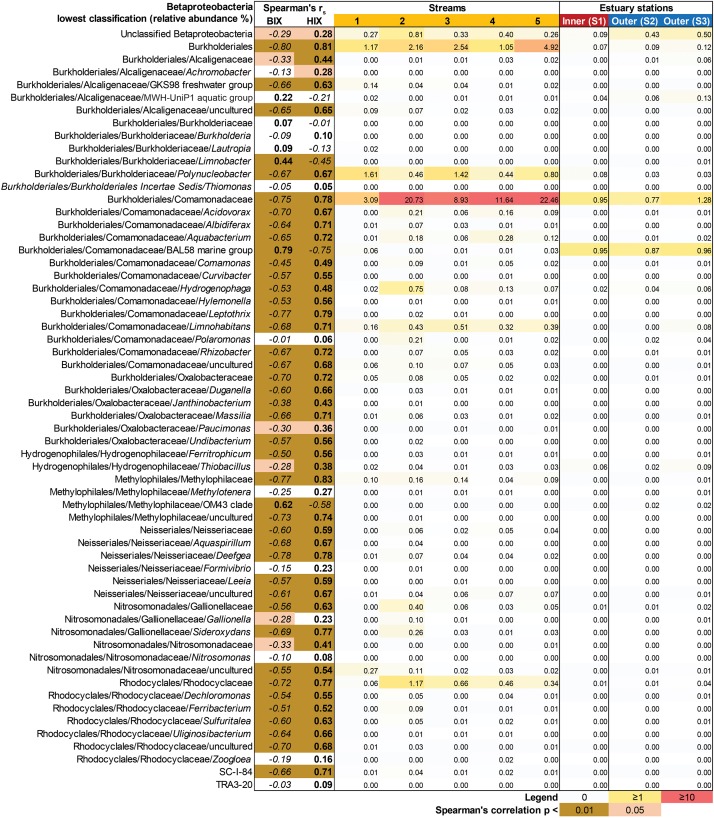
Heatmap showing all Betaproteobacteria OTUs grouped into the lowest classifications (i.e., order, family, or genus). The heatmap (yellow–red color gradient) shows the relative abundance (%) of the whole prokaryotic community, with white to yellow (0–1%), yellow to red (1–10%), and red (≥10%). The columns show the average relative abundances of the samples from each study system (i.e., five streams and 1 m depth data used from the three stations in the estuary). The FDOM biological index BIX (i.e., autochthonous origin) and humification index HIX (i.e., allochthonous origin) were used in Spearman correlations with the Betaproteobacteria groups. The results are indicated in the columns BIX and HIX with brown colors (*p* < 0.01) and light brown (*p* < 0.05). The italic text denotes negative correlations and bold text denotes positive correlations.

**FIGURE 8:**
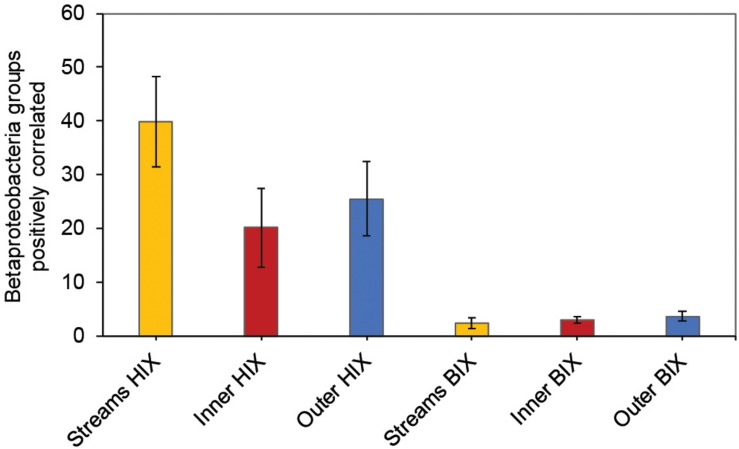
Amount of Betaproteobacteria groups (lowest taxonomic classifications as shown in Figure 7) that correlated positively with either the HIX or BIX index. The error bars show one standard deviation, streams *n* = 27, inner estuary (station 1, 1 m depth data) *n* = 7, outer estuary (stations 2 and 3, 1 m depth data) *n* = 16.

In contrast to the estuary sites, the microbial community composition in the streams was more associated with humic indicators with positive correlations for HIX and peak C, while correlating negatively with indicators of autochthonous carbon (S_275__–__295_ and BIX) (Figure 6). This included the taxa Lentisphaerae, Gemmatimodetes, Fibrobacteres, Beta-, Delta-, and Epsilonproteobacteria (Figure 5C). In addition, some phyla correlated positively to nutrients including, e.g., Chloroflexi, Chlorobi, and Acidobacteria. The Bacteroidetes correlated negatively with TN but positively with conductivity (Figure 5C) while Actinobacteria correlated positively with DOC:DON (Figure 5C). Looking closer at the top 30 OTUs with a statistical significance in the streams, fewer correlated significantly with nutrients than in the estuary (Figure 6). Among the most abundant OTUs (totaling 4.5% of the relative abundance), the Betaproteobacteria order Burkholderiales were positively correlated with the humic-like indicator peak C (Figure 6). While OTUs belonging to the Betaproteobacteria family Comamonadaceae and Epsilonproteobacteria genus *Arcobacter* were positively correlated with either peak C or HIX while being negatively correlated with the S_275__–__295_ slope and the biological index BIX (Figure 6). Moreover, most of the top abundant OTUs correlating with humic-like DOM did not correlate with nutrient or abiotic variables (Figure 6). Finally, ∼60% of phyla had significant but weak correlations (*r*_s_ < 0.7 o*r* > −0.7) with humic-like indicators in the streams (Supplementary Table S5). The majority of the Betaproteobacteria on the lowest classified taxonomic level correlated positively with the HIX and negatively with the biological index BIX (Figure 7), with unclassified OTUs belonging to the family Comamonadaceae having the highest relative abundance (Figure 7). A BLAST search against the NCBI NT database showed that the top abundant OTUs belonging to the family Comamonadaceae (OTUs 66 and 103; Figure 6) showed that OTU 66, which correlated positively with HIX (Figure 6), had the best database hit to *Malikia granosa* (99.14% identity, *e*-value = 0.0, score = 837). The best hit in the database for OTU 103, which did not correlate with DOM characteristics (Figure 6), was the genus *Limnohabitans* (99.35% identity, *e*-value = 0.0, score = 843). The number of Betaproteobacteria groups (at the lowest taxonomic classification) that correlated positively with HIX was significantly higher in the streams when compared to the inner and outer estuary (one-way ANOVA tests, *F* = 31.7 and 32.9, *p* < 0.01 for both, respectively; Figure 8). In contrast, the amount of positive correlations with Betaproteobacteria groups and BIX was higher in the outer estuary compared to the streams (one-way ANOVA, *F* = 21.8, *p* < 0.01; Figure 8).

## Discussion

Different microbial taxa are associated with either humic-like allochthonous versus autochthonous DOM (Roiha et al., 2016). In this study, the number of correlations by Betaproteobacteria populations with humic-like DOM (HIX index) decreased from the terrestrial streams to the estuary (Figure 8). Even though salinity is known to strongly influence the microbial community composition (Fortunato et al., 2012), the multiple regression analysis indicated that humic-like DOM was one of the main drivers influencing the relative abundance of populations belonging to Betaproteobacteria (Table 1 and Figure 7). As the streams discharged into the higher saline estuary the prokaryotic community composition abruptly changed (Figure 2B). This stark shift in the relative abundance of OTUs at the stream and marine stations indicated that as DOM travels through the land-to-sea interface different bacterial guilds, in addition to Betaproteobacteria, continuously degrade it. Climate change is expected to increase river discharge (van Vliet et al., 2013) that will likely deliver more allochthonous DOM to the coastal microbiome (Kritzberg and Ekström, 2012). This will also enrich the estuary with nutrients, such as nitrogen and phosphorus, that can sustain phytoplankton blooms and subsequent increased production of autochthonous DOM (Asmala et al., 2018b). These two contrasting processes are likely to change the DOM distribution and characteristics over time in coastal systems.

The concentration of DOC was higher (Supplementary Table S1A) and DOM was characterized as humic-like in the streams (Supplementary Table S1B) (Asmala et al., 2018b) due to humic matter leaching from terrestrial landscapes (Massicotte et al., 2017). Accordingly, we observed a strong correlation between the microbial community composition and the mainly terrestrial-like characteristics of the DOM pool in the streams. This suggested the community was adapted to degrade this colored source of carbon and it thereby determined the structure of the stream microbial community compositions. In the inner estuary, approximately 50% of the taxa (dominated by Proteobacteria) were more closely associated with allochthonous DOM (Figure 4B). It has previously been shown in the laboratory that marine and estuary microbial communities fed with humic substances become dominated by Alpha- and Gammaproteobacteria (Rocker et al., 2012). Amaral et al. (2016) sampled a subtropical lagoon and used catalyzed reporter deposition fluorescence *in situ* hybridization (CARD-FISH) to find that Betaproteobacteria were partly associated with both allochthonous and autochthonous DOM. In addition, Betaproteobacteria have been shown in the laboratory to be favored upon enrichment with high molecular weight DOM (Sosa et al., 2015), but not when fed with humic acids (Rocker et al., 2012). However, as Betaproteobacteria correlated positively with CDOM and FDOM (Figure 3), and were significantly associated with the HIX index (Table 1), they likely degraded allochthonous DOM. The data showed that especially unclassified sequences belonging to the Betaproteobacteria family Comamonadaceae were associated with humic-like DOM (Figure 7), with the genus *M. granosa* belonging to one of the top abundant unclassified OTUs. This is an obligatory aerobic, heterotrophic, and nitrate reducing bacteria that has previously been isolated from activated sludge at a wastewater treatment plant (Spring et al., 2005). The second inner estuary microbial guild (dominated by Actinobacteria and Cyanobacteria) constituting the second 50% of the relative community composition was more associated with abiotic variables and nutrients (Figures 2, 3, 4B). This was also supported by a large portion of the relative abundance of Actinobacteria that strongly negatively correlated with humic-like DOM (Figure 6) and Cyanobacteria being significantly associated with temperature in the regression analysis (Table 1). Especially OTUs classified to the Actinobacteria genus Candidatus *Aquiluna* had a high relative abundance in the estuary dataset (Figures 2B, 6). A BLAST search resulted only in uncultured bacterium clones. However, based on one available genome sequence this genus is known to contain at least one species suggested to be a photoheterotroph (Kang et al., 2012), which could explain the negative correlation to humic-like DOM characteristics. In addition, Actinobacteria was mainly associated with salinity (Table 1 and Figure 3) as has been previously reported along the salinity gradient in the Baltic Sea (Herlemann et al., 2011). Finally, microbial populations belonging to Alphaproteobacteria have been observed to be specialized to degrade algal matter (Teeling et al., 2012) and results from this study found a correlation (possibly indirectly related to the salinity gradient) between Alphaproteobacteria and the BIX index when tested for all data from the marine and stream stations (Figure 3). That the inner estuary microbial community had less obvious associations with either only allochthonous or only autochthonous DOM (and other abiotic variables) was also indicated with the CCA axes having no statistical significance (compared to the outer estuary and streams). Based on *in situ* data over a 6-month time series and coupling molecular data to a large amount of optical DOM variables, the results show that distinct components of the bacterial community were associated with organic carbon originating from streams and that the community in the inner estuary were indicated to be associated with both DOM sources and nutrients. These findings suggest that coastal ecosystems are essential zones of DOM degradation and on a global scale. These zones likely prevent large amounts of allochthonous DOM from reaching the open sea.

In the outer estuary, the relationship between abiotic factors and microbial community composition based on the CCAs (Figure 4A), spearman correlations (Figure 5A), and the abundant OTUs (Figure 6) suggested that the major factors explaining the microbial community composition were nutrients and salinity. For instance, Bacteroidetes were partly associated with, e.g., temperature and nutrients in the estuary and streams (Figure 5). Hutalle-Schmelzer et al. (2010) sampled lake water and conducted a laboratory study using denaturing gradient gel electrophoresis (DGGE) and found specific populations of Bacteroidetes to be favored after addition of humic substances. Considering our study used field samples over multiple seasons, it is possible that temperature, pH, conductivity, and nutrients were more important drivers for the relative abundance of Bacteroidetes and therefore, this phylum was not apparently associated with CDOM (Figures 2, 3). Furthermore, compared to DGGE we used 16S rRNA gene amplicon sequencing that likely gave a better representation of the whole microbial community present in the estuary. However, the primers used were not modified for SAR11 bacteria (Apprill et al., 2015) and these populations would therefore be underestimated in the study. These findings suggested a transition in the microbial community had occurred from an allochthonous supported stream, an allochthonous and autochthonous plus other abiotic variables supported inner estuary, to a marine zone more dependent on nutrient, salinity, and temperature availability.

The inner estuary of Roskilde Fjord has an average residence time of 8 months (Kamp-Nielsen, 1992) and a large portion of terrestrial DOM is suggested to be lost through microbial degradation before reaching the outer estuary (Asmala et al., 2018b). However, it is unknown how changes in the coastal microbiome will affect degradation of the DOM pool. Our findings demonstrate that both major bacterial groups and specific OTUs were associated with either allochthonous or autochthonous DOM and abiotic variables. This *in situ* field study supports that the findings from a broad range of laboratory studies are at least partially relevant to inform about key processes that occur in coastal systems (Logue et al., 2015; Sosa et al., 2015; Traving et al., 2017). Osterholz et al. (2018) found that bacterial community structure in a large coastal estuary (Delaware Estuary, United States) were partly explained by the molecular composition of DOM (based on mass spectrometry data). Here we build further on this knowledge by (1) studying a smaller coastal estuary more prone to be affected by allochthonous DOM from terrestrial runoff; (2) including adjacent streams; (3) using a comprehensive set of nutrients and optical DOM variables; and (4) sampling points with a higher time resolution. Coastal zones are critical meeting points for allochthonous and autochthonous DOM (Massicotte et al., 2017; Asmala et al., 2018b) and collectively these findings imply that changes in microbial community composition at the land–sea border have a particularly strong influence on DOM transformations.

Temperature is an important factor that modulates microbial population dynamics and process rates. In the present data set, cyanobacteria had a strong positive correlation with temperature (Figure 3), and a significant association in the regression analysis (Table 1), while inner estuary humic-like DOM-associated microbial groups were negatively correlated (Figure 5B). In general, temperature is a good predictor for cyanobacterial biomass (Beaulieu et al., 2013) and our data support the expectation that continued climate warming will favor a community shift to cyanobacteria (Paerl and Huisman, 2008). This could be especially important considering that autochthonous labile DOM in Roskilde Fjord has been shown to potentially be processed into more recalcitrant protein-like DOM (Asmala et al., 2018b). A further increase in cyanobacteria and decrease in humic-like associated microbial phyla in coastal transition zones are therefore expected to increase the portion of autochthonous DOM. In the current study, we investigated microbial communities associated with DOM characteristics. However, in addition to microbial degradation of DOM, physical factors such as photochemical mineralization by light also influences DOM composition and availability (Koehler et al., 2016). Future changes in DOM characteristics are therefore not solely dependent on the microbial community.

## Conclusion

In conclusion, the results show that the microbial community composition in the outer estuary (closer to the open sea) was largely associated with salinity and nutrients, while the inner estuary formed two clusters linked to either nutrients and autochthonous DOM or allochthonous DOM characteristics. In contrast, humic-like DOM was found to strongly influence the microbial community structure in the streams. Especially the Betaproteobacteria family Comamonadaceae had a strong association with allochthonous DOM in the streams and estuary system. In contrast, distinct populations were more associated with abiotic variables such as salinity (Actinobacteria family Microbacteriaceae) or temperature (Cyanobacteria genus *Synechococcus*). Furthermore, the stark shift in the relative abundance of OTUs between stream and marine stations indicates that as DOM travels through the land-to-sea interface, different bacterial guilds continuously degrade it. These communities control carbon cycling in coastal ecosystems, where important changes in DOM composition are predicted to occur with climate change (Asmala et al., 2018b). Therefore, these findings suggest that in coastal zones specific microbial populations associated with either autochthonous or allochtonous DOM will have an increased role in coastal carbon cycling.

## Data Availability Statement

The datasets generated for this study can be found in the NCBI BioProject id: PRJNA396662.

## Author Contributions

EB conducted the molecular laboratory work, bioinformatics, analyzed the data, and drafted the manuscript. EA and JC gave feedback on the manuscript. EA sampled in the field and helped in the laboratory to filter and store DNA samples. JC assisted in the design of the study. JP and MD helped draft the manuscript. JP designed the study. MD assisted in the design of the study. All authors gave the final approval for the publication.

## Conflict of Interest

The authors declare that the research was conducted in the absence of any commercial or financial relationships that could be construed as a potential conflict of interest.
