# Supplementary figures and images for: Distinct Coastal Microbiome Populations Associated With Autochthonous- and Allochthonous-Like Dissolved Organic Matter
Source: Front Microbiol. 2019 Nov 7;10:2579. doi: 10.3389/fmicb.2019.02579 (PMC6854034; doi:10.3389/fmicb.2019.02579)

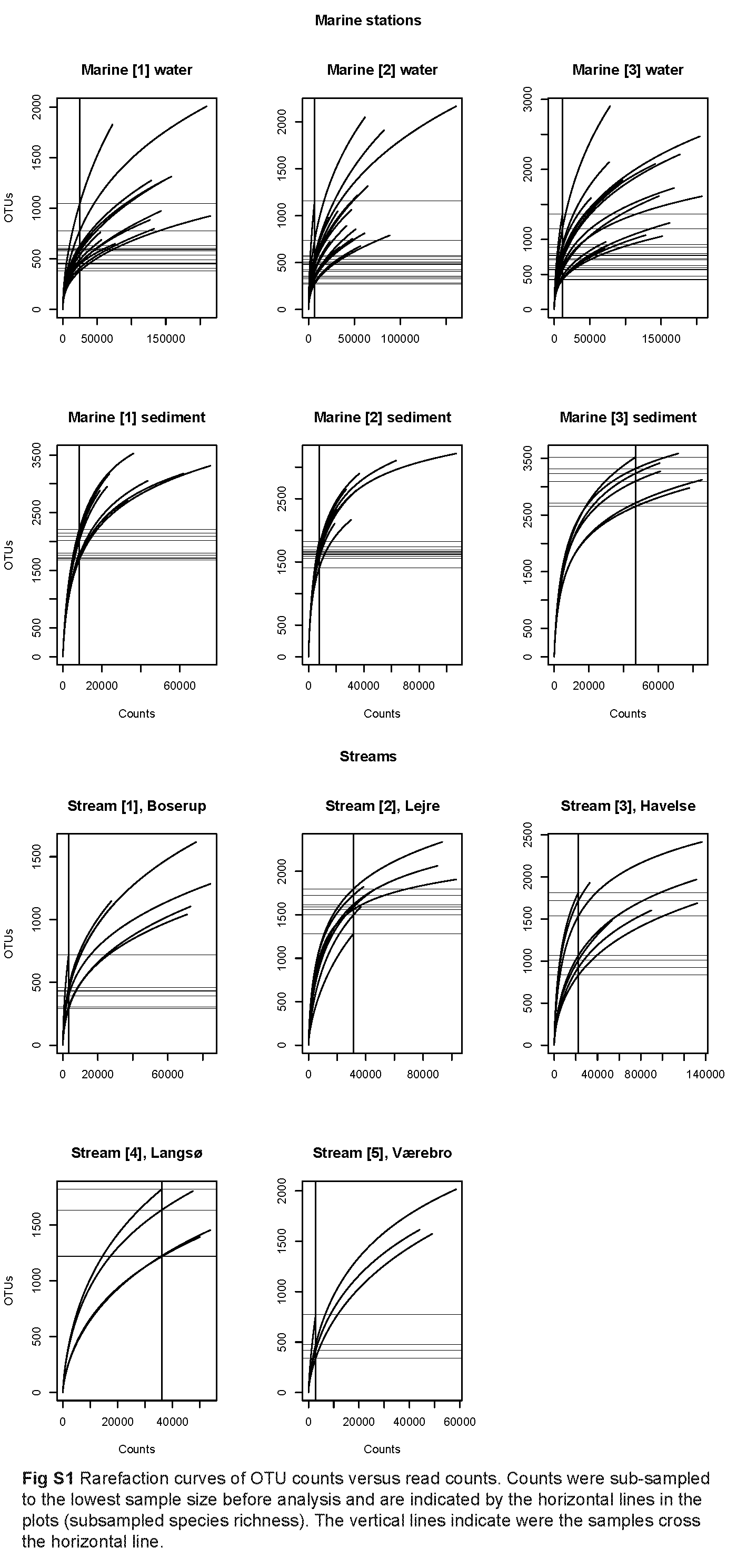

Supplement: Supplementary file 1 [file Image_1.PNG]

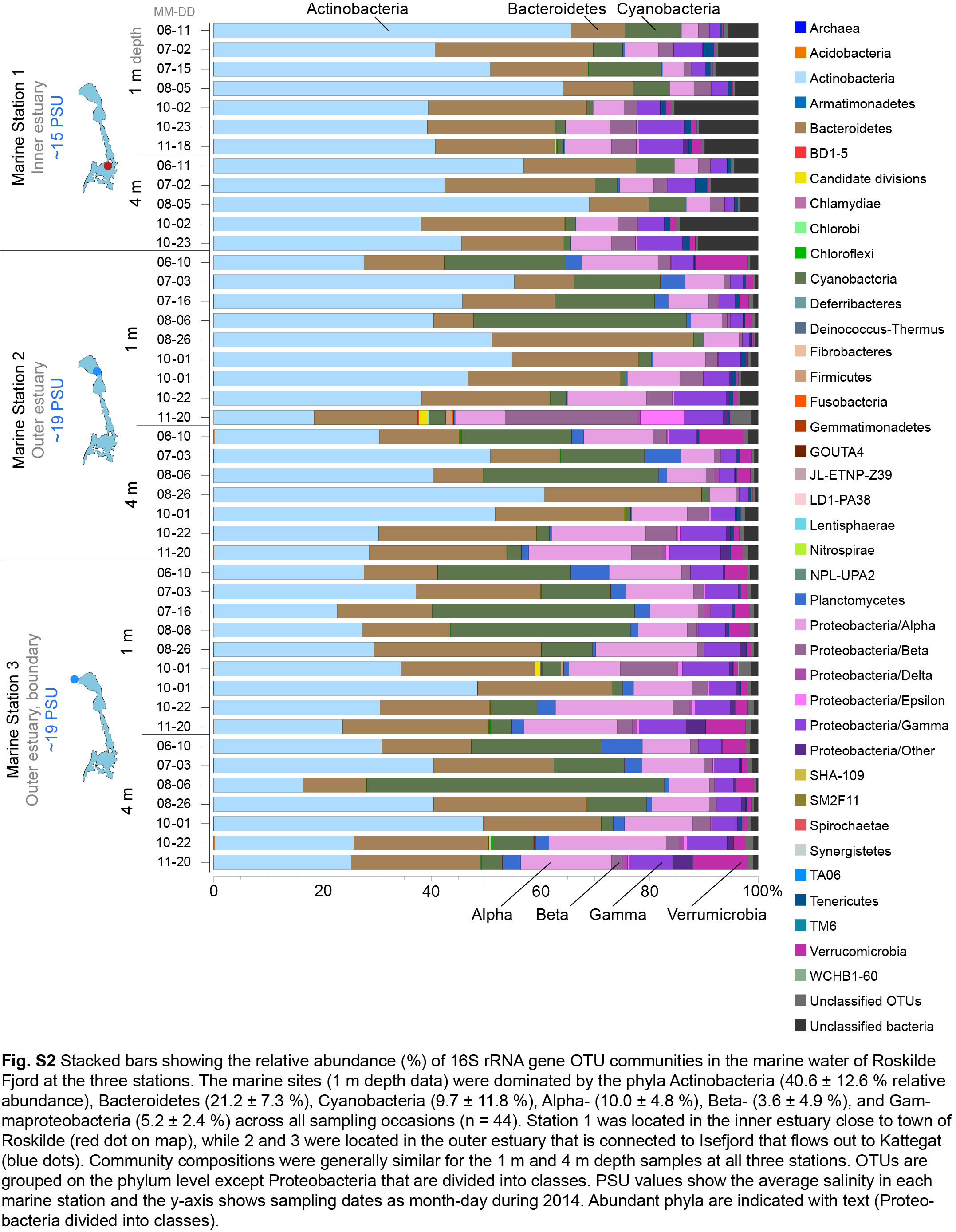

Supplement: Supplementary file 2 [file Image_2.PNG]

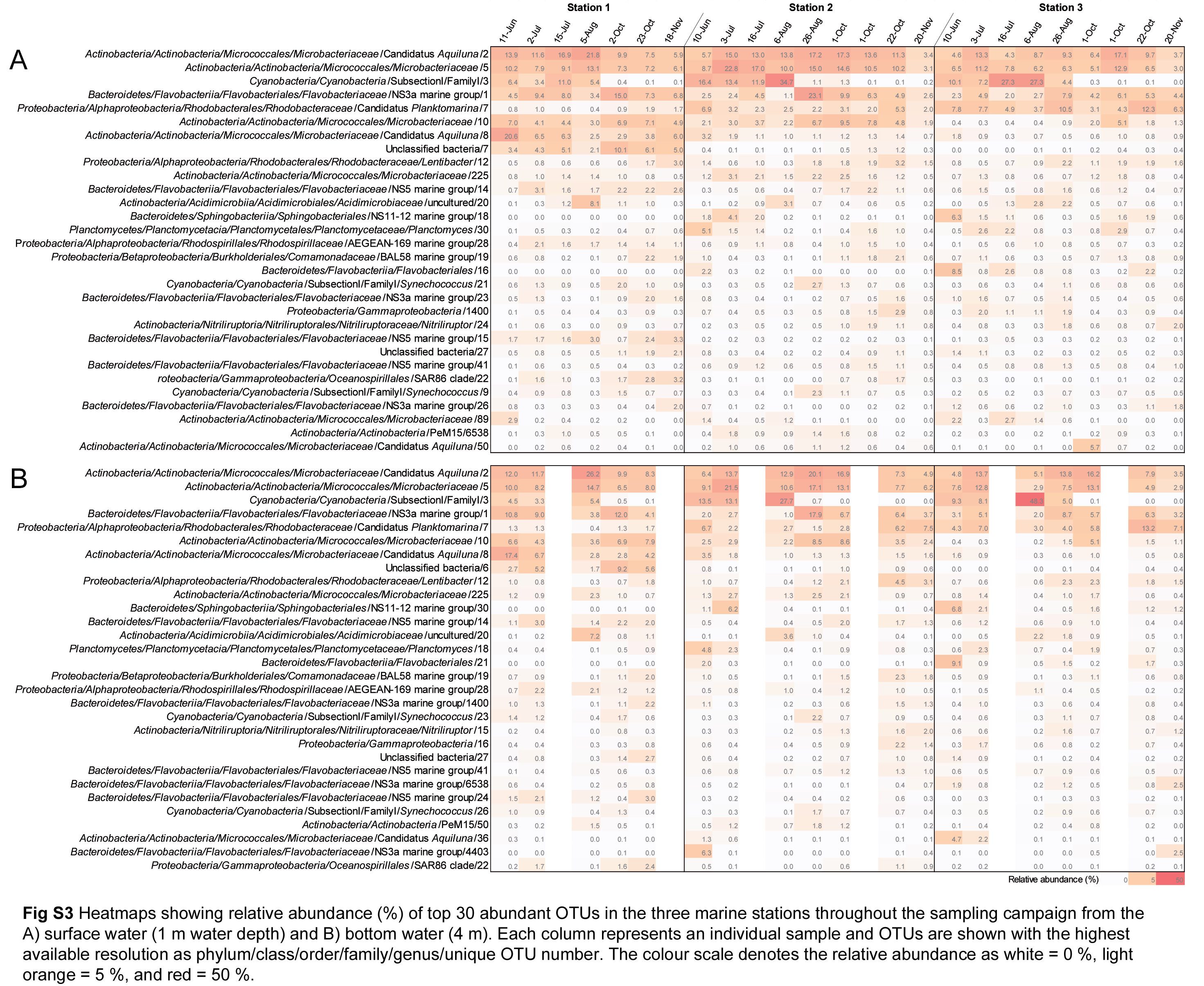

Supplement: Supplementary file 3 [file Image_3.PNG]

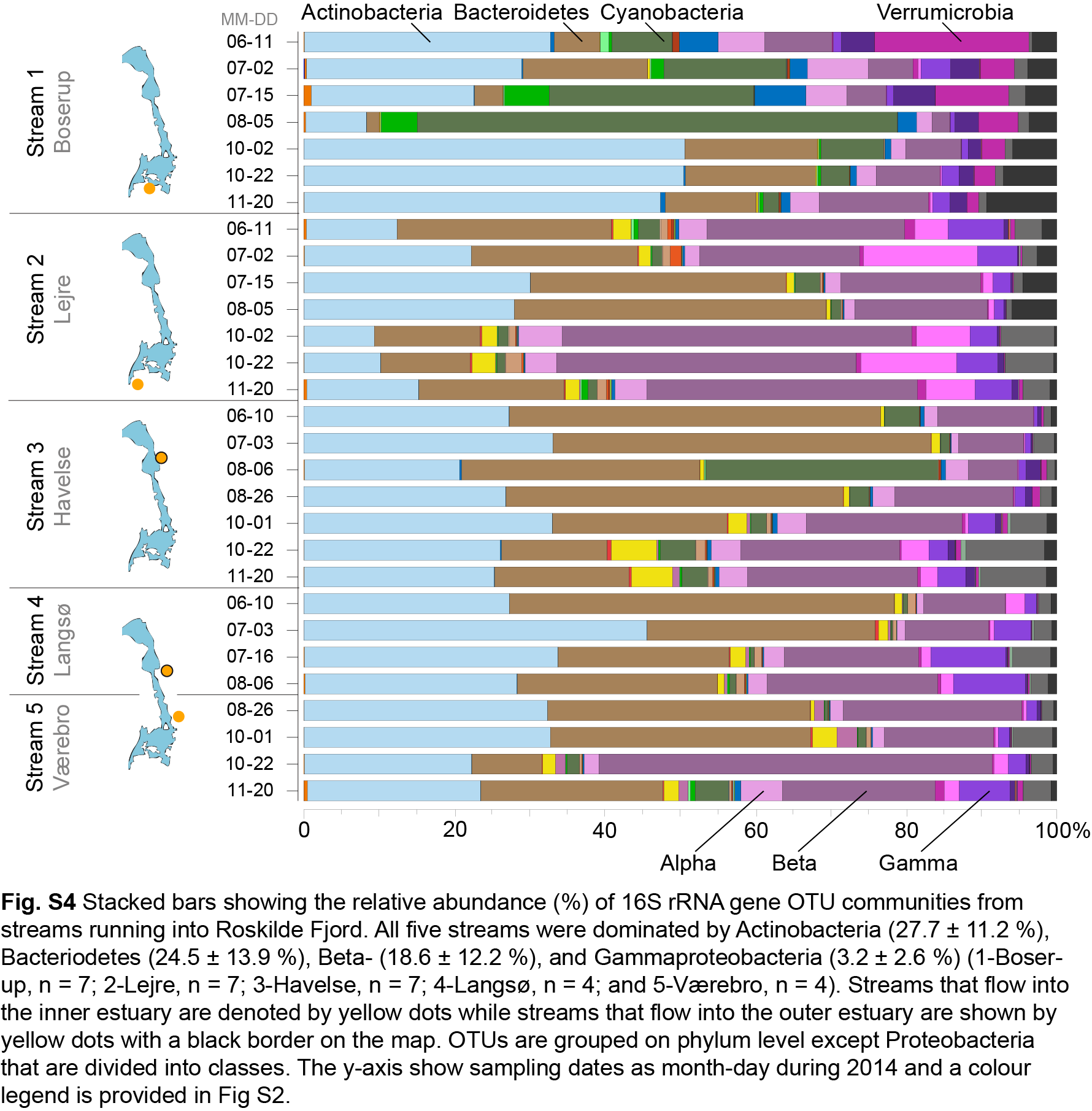

Supplement: Supplementary file 4 [file Image_4.PNG]

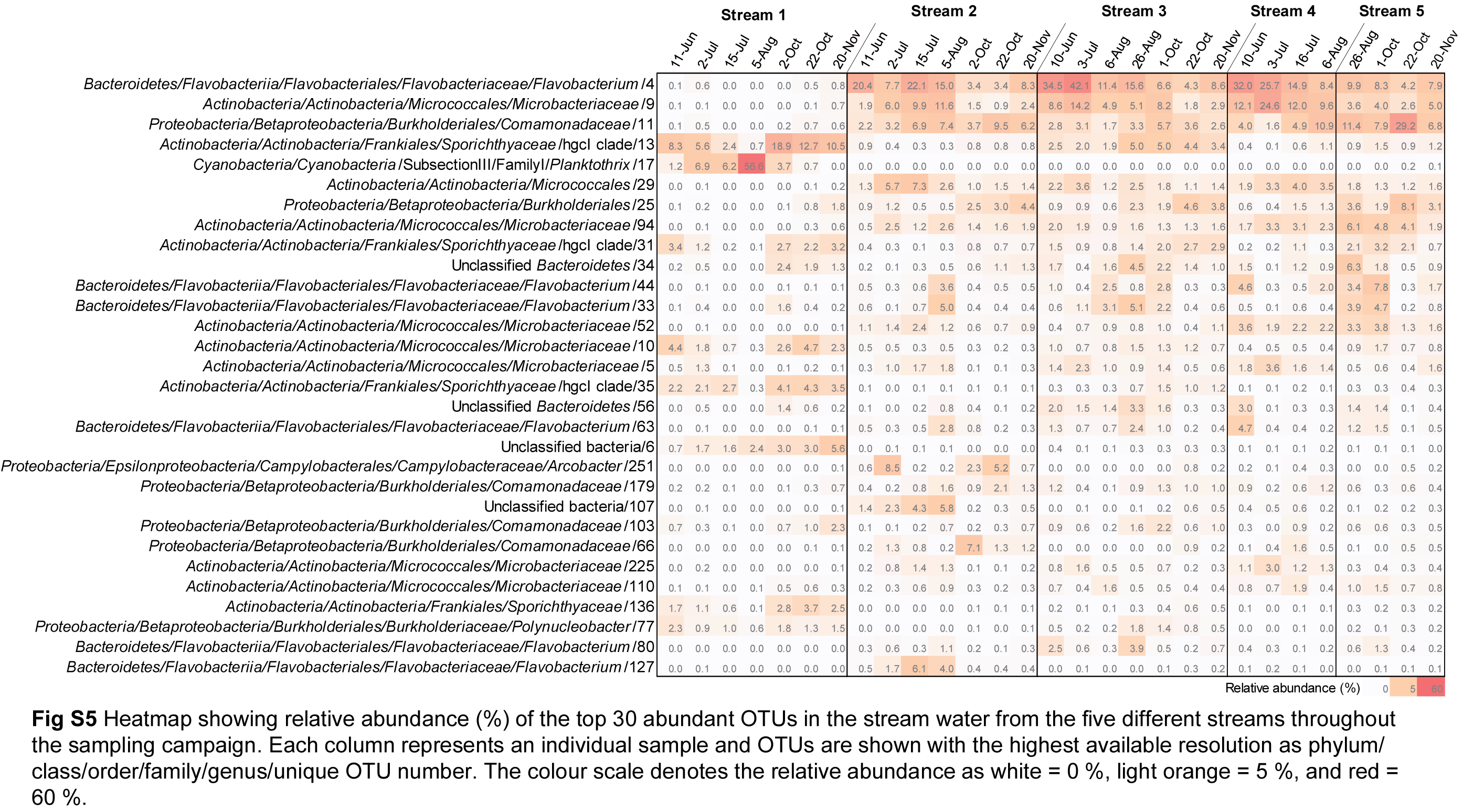

Supplement: Supplementary file 5 [file Image_5.PNG]
