# Supplementary material for: Distinct Coastal Microbiome Populations Associated With Autochthonous- and Allochthonous-Like Dissolved Organic Matter
Source: Front Microbiol. 2019 Nov 7;10:2579. doi: 10.3389/fmicb.2019.02579 (PMC6854034; doi:10.3389/fmicb.2019.02579)
Supplement: Supplementary file 6 [file Table_1.DOCX]

**Table S1A** Chemistry parameters measured in the ‘outer estuary’ (*n* = 31) and ‘inner estuary’ (*n* = 12) of Roskilde Fjord between June 10 and November 22 2014, typically once or twice per month. In addition, five streams flowing into Roskilde Fjord were sampled (*n* = 27) (Full details and data from: E. Asmala et al. (1)). Values for Salinity (estuary only) and conductivity (streams only) are shown as PSU and µS cm^-1^, respectively. The remaining parameters: dissolved inorganic nitrogen and phosphorous (DIN and DIP), dissolved organic nitrogen and carbon (DON and DOC), as well as total nitrogen and phosphorous (TN and TP) are all shown in µM, mean ± one standard deviation.

| **Site** | **Temp** | **pH** | **Salinity/ Cond.** | **DIN** | **DIP** | **DON** | **DOC** | **TN** | **TP** |
| --- | --- | --- | --- | --- | --- | --- | --- | --- | --- |
| Outer estuary | 17 ± 5 | 8.2 ± 0.2 | 19 ± 1 | 3 ± 3 | 1 ± 1 | 21 ± 17 | 351 ± 61 | 31 ± 10 | 2 ± 1 |
| Inner estuary | 18 ± 5 | 8.3 ± 0.2 | 15 ± 0 | 4 ± 3 | 7 ± 2 | 12 ± 6 | 524 ± 45 | 48 ± 14 | 8 ± 2 |
| Streams | 16 ± 4 | 8.0 ± 0.5 | 656 ± 133 | 77 ± 41 | 1 ± 1 | 3 ± 4 | 702 ± 131 | 129 ± 51 | 5 ± 3 |

**Table S1B** Optical parameters for CDOM and FDOM measured in the ‘outer estuary’ and ‘inner estuary’ of Roskilde Fjord, as well as the streams (Full details and data from: E. Asmala et al. (1)). CDOM parameters included aromatic indicators (*a*_(CDOM254)_ m^-1^, *a*_(CDOM400)_ m^-1^, SUVA_254_ m^2^ g^-1^ C) and low molecular weight indicators (spectral slopes for S_275-295_ µm^-1^ and S_350-400_ µm^-1^). FDOM parameters included the humic-like indicator (Peak C R.U.), humification index HIX (i.e. allochthonous origin), protein-like labile indicator (Peak T R.U.), and the biological index BIX (i.e. autochthonous origin), mean ± one standard deviation.

| **Site** | ***a* _(CDOM254)_** | ***a* _(CDOM400)_** | **SUVA_254_** | **S_275-295_** | **S_350-400_** | **Peak C** | **HIX** | **Peak T** | **BIX** |
| --- | --- | --- | --- | --- | --- | --- | --- | --- | --- |
| Outer estuary | 20 ± 4 | 2 ± 1 | 2.1 ± 0.3 | 23 ± 2 | 13 ± 6 | 0.1 ± 0.0 | 6.2 ± 1.6 | 0.1 ± 0.0 | 0.8 ± 0.0 |
| Inner estuary | 36 ± 1 | 3 ± 0 | 0.0 ± 0.0 | 22 ± 1 | 17 ± 2 | 0.3 ± 0.0 | 8.4 ± 1.7 | 0.2 ± 0.0 | 0.8 ± 0.0 |
| Streams | 66 ± 18 | 6 ± 2 | 3.2 ± 0.4 | 16 ± 1 | 18 ± 1 | 1.3 ± 0.5 | 19.9 ± 9.3 | 0.3 ± 0.1 | 1.3 ± 3.8 |

**References**

1. Asmala E, Haraguchi L, Markager S, Massicotte P, Riemann B, Staehr PA, Carstensen J. 2018. Eutrophication leads to accumulation of refractory autochthonous organic matter in coastal environment. Global Biogeochemical Cycles 32:1673-1687.
